# An evolutionarily conserved negative feedback mechanism in the Hippo pathway reflects functional difference between LATS1 and LATS2

**DOI:** 10.18632/oncotarget.8211

**Published:** 2016-03-19

**Authors:** Gun-Soo Park, Hyangyee Oh, Minchul Kim, Tackhoon Kim, Randy L. Johnson, Kenneth D. Irvine, Dae-Sik Lim

**Affiliations:** ^1^ National Creative Research Center for Cell Division and Differentiation, Department of Biological Sciences, Korea Advanced Institute of Science and Technology (KAIST), Daejeon, South Korea; ^2^ Howard Hughes Medical Institute, Waksman Institute and Department of Molecular Biology and Biochemistry, Rutgers University, Brunswick, New Jersey, USA; ^3^ Department of Cancer Biology, University of Texas, M.D. Anderson Cancer Center, Houston, Texas, USA

**Keywords:** Hippo pathway, YAP, LATS2, negative feedback, homeostasis

## Abstract

The Hippo pathway represses YAP oncoprotein activity through phosphorylation by LATS kinases. Although variety of upstream components has been found to participate in the Hippo pathway, the existence and function of negative feedback has remained uncertain. We found that activated YAP, together with TEAD transcription factors, directly induces transcription of LATS2, but not LATS1, to form a negative feedback loop. We also observed increased mRNA levels of Hippo upstream components upon YAP activation. To reveal the physiological role of this negative feedback regulation, we deleted *Lats2* or *Lats1* in the liver-specific Sav1-knockout mouse model which develops a YAP-induced tumor. Additional deletion of *Lats2* severely enhanced YAP-induced tumorigenic phenotypes in a liver specific *Sav1* knock-out mouse model while additional deletion of *Lats1* mildly affected the phenotype. Only *Sav1* and *Lats2* double knock-down cells formed larger colonies in soft agar assay, thereby recapitulating accelerated tumorigenesis seen *in vivo*. Importantly, this negative feedback is evolutionarily conserved, as Drosophila Yorkie (YAP ortholog) induces transcription of Warts (LATS2 ortholog) with Scalloped (TEAD ortholog). Collectively, we demonstrated the existence and function of an evolutionarily conserved negative feedback mechanism in the Hippo pathway, as well as the functional difference between LATS1 and LATS2 in regulation of YAP.

## INTRODUCTION

The Hippo pathway is an evolutionarily conserved developmental and tumor-suppressive signaling pathway which controls proliferation and differentiation of cells. Originally discovered in Drosophila, the Hippo core components consist of several kinases and scaffold proteins including MST1 and −2 (MST1/2, mammalian orthologs of Hippo in Drosophila), SAV1 (mammalian ortholog of Salvador in Drosophila), LATS1 and −2 (LATS1/2, mammalian orthologs of Warts in Drosophila), and MOB1A and –B (MOB1A/B, mammalian orthologs of Mats in Drosophila). Activation of MST1/2 causes phosphorylation of LATS1/2 with the help of SAV1, then LATS1/2 become fully activated through MOB1A/B binding induced autophosphorylation. Activated LATS1/2 then phosphorylate and inactivate their target proteins, YAP(Yes-associated protein) and its paralog TAZ(transcriptional co-activator with PDZ-binding motif) [1–3].

YAP and TAZ (YAP/TAZ), orthologs of Yorkie in Drosophila, are oncogenes which exert a transforming effect in cell lines and induce tumorigenesis in transgenic mouse models [4–6]. YAP/TAZ binds to several transcription factors, including TEAD/TEF (TEAD TFs), and function as transcriptional co-regulator to activate or repress transcription of target genes [7–9].

Ablated or uncontrolled YAP activity results in developmental or tumorigenic defects in various organs, even from the preimplantation embryo stage [10–12]. Therefore, a variety of cues including humoral factors, cytoskeleton conformations, mechanical forces and energy status can affect the Hippo pathway and YAP/TAZ [13–19]. In Drosophila, Expanded, Merlin and Kibra had been identified as upstream components which convey initial signals to the Hippo core components [20].

Negative feedback mechanism in a signaling pathway maintains homeostasis of output effects. The presence of negative feedback in the Hippo pathway has been suggested in early Drosophila studies, showing that Expanded, Merlin, and Kibra are targets of Yorkie [21, 22]. In addition, our group previously showed that Hippo pathway component proteins are increased in the *Sav1* knock-out mouse model [23]. However, it is unclear whether this induction of upstream components of the Hippo pathway is conserved and functional in mammalian cells.

In this study, we reveal that YAP up-regulates the expression of its direct suppressor LATS2 at the transcriptional level by a time-course analysis using an inducible system. Such up-regulation of LATS2 was a consequence of direct binding of the YAP/TEAD complex to the promoter region of *LATS2*. Moreover, additional deletion of *Lats2* in liver-specific *Sav1* knock-out mouse model accelerated tumorigenesis, confirming the existence of a bona fide negative feedback regulation of YAP through LATS2. Notably, this negative feedback regulation of YAP is evolutionarily conserved, as Drosophila Yorkie induces the transcription of Warts.

## RESULTS

### YAP induces transcription of LATS2

**Figure 1:**
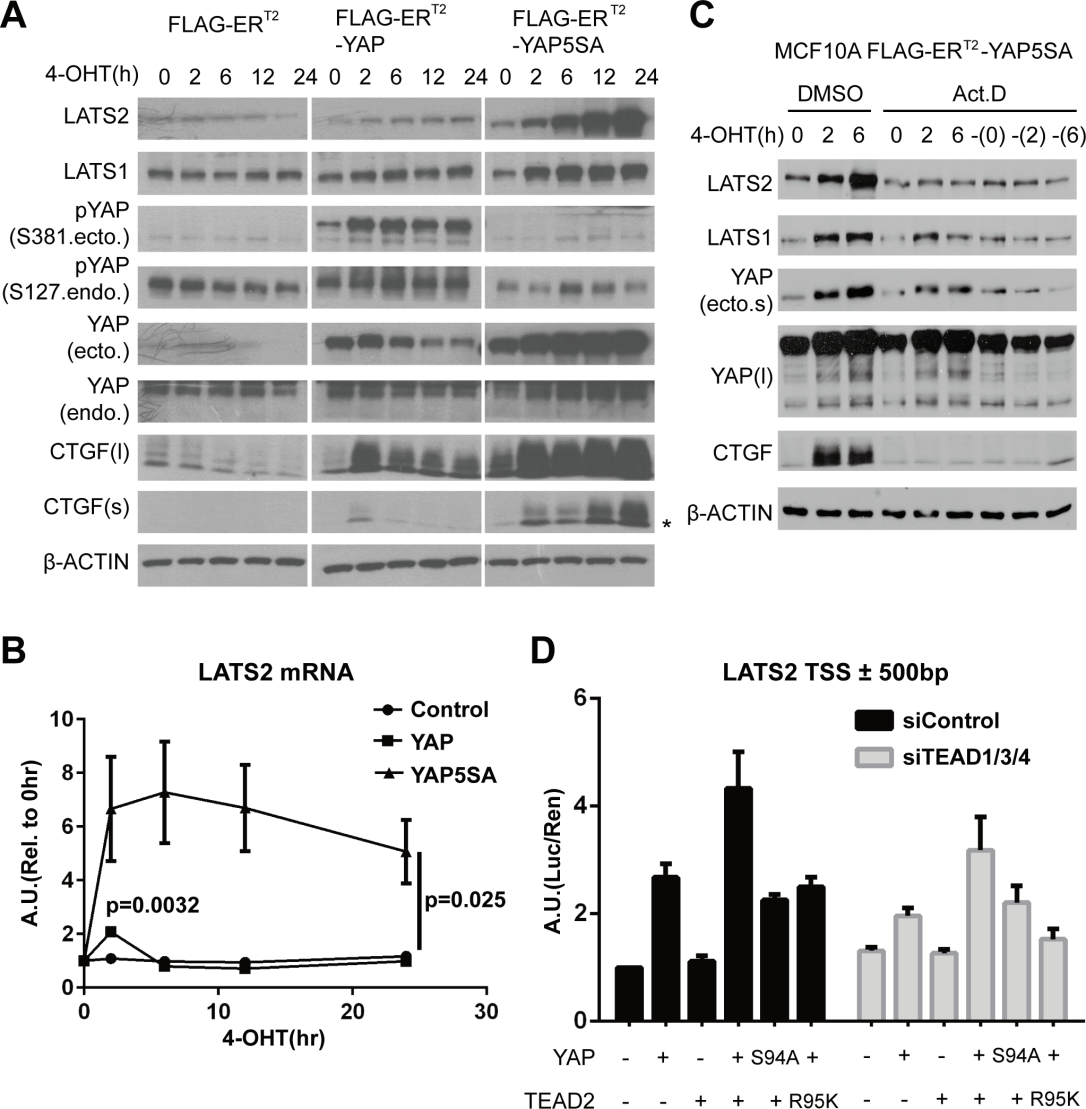
YAP activation directly induces LATS2 transcription (**A** and **B**) LATS2 expression levels were increased by YAP activation. MCF-10A cells expressing the indicated constructs were treated with 4-OHT for up to 24 hours. LATS2 up-regulation was demonstrated by Western blot (A) and qRT-PCR (B). Asterisk in the CTGF blot indicates non-specific bands. *p*-values from ANOVA among three cell lines and from two-tailed *t*-test for wild-type YAP induced sample at 2-hour time point are indicated in panel (B). (**C**) MCF-10A cells expressing 4-OHT–inducible YAP5SA were pre-treated with actinomycin D for 30 minutes and then with 4-OHT for 0, 2, and 6 hours. The dash (‘–’) in the right-most three lanes indicates 4-OHT–untreated samples harvested at the same time as other samples. (**D**) Luciferase reporter assay using a *LATS2* promoter region. HEK-293T cells were transfected with the indicated constructs, and luciferase activity was measured as the ratio of firefly (experimental) luciferase to Renilla (control) luciferase.

To investigate potential negative feedback mechanisms in the Hippo pathway, we employed a tamoxifen-inducible expression system in which YAP activity can be acutely induced, enabling us to distinguish direct impacts of YAP activation from indirect consequences. Expression of wild-type YAP or constitutively active YAP5SA mutant, whose five serine residues in consensus motifs for LATS1/2 are substituted to alanine [24], was induced in MCF-10A cells by treatment with 4-hydroxytamoxifen (4-OHT), and expression levels of Hippo pathway components were measured. Acute induction of wild-type YAP transiently increased the expression of CTGF and CYR61, whereas induction of YAP5SA constitutively up-regulated those genes (Figure 1A, Figure S1A). This specific pattern implies the presence of negative feedback on YAP activity and its dependency on YAP phosphorylation.

Interestingly, continuous YAP activation dramatically increased the expression of LATS2 (Figure 1A), a direct upstream regulator of YAP, and similarly increased T1079- and S909- phosphorylated forms of LATS (Figure S1B). However, it had no effect on MST1/2 and SAV1 (Figure S1B). LATS2 up-regulation by YAP was also associated with a reduction in TAZ protein levels (Figure S1B). We further confirmed up-regulation of LATS2 and phosphorylated form of LATS1/2 in the NMuMG and HaCaT cell lines (Figure S1C). Induced expression of YAP2SA and TAZ2SA mutants – S127 and S381 for YAP and corresponding sites for TAZ in which control cytoplasmic translocation and degradation through phosphorylation by LATS1/2 are mutated – also up-regulated LATS2 protein levels, thus the phenomenon is common to YAP and TAZ (Figure S1D).

Since YAP/TAZ are transcriptional co-regulators, we determined whether LATS2 transcription is up-regulated by measuring its mRNA levels. As expected, acute induction of wild-type YAP transiently increased the *LATS2* mRNA levels (Figure 1B), similar to its effects on *CTGF* and *CYR61* mRNA (Figure S1A). Treatment of transcription inhibitor actinomycin-D blocked LATS2 induction (Figure 1C). Similarly, expression of a dominant negative form of YAP lacking C-terminal transactivation domain did not increase LATS2 protein levels compared to YAP5SA (Figure S3A). Thus, transcriptional activation is necessary for LATS2 induction by YAP. Finally, luciferase reporter assay using a promoter region of *LATS2* showed that reporter signal intensity correlated with YAP activity (Figure 1D). YAP activity was confirmed to exert no or negligible effect on translational or post-translational control of control of LATS2 (Figure S3B and S3C). Taken together, these results indicate that YAP/TAZ activation induces transcription of *LATS2*.

### Some Hippo upstream components are regulated by YAP activity

Whereas induction of LATS2 expression was prominent in Western blot analyses of cell lines harboring inducible wild-type YAP or YAP5SA mutant expression constructs, other Hippo components, such as KIBRA, also showed a pattern of increasing expression in response to YAP activation (Figure S1B and S1C). Because our ChIP-seq analyses also revealed additional peaks near Hippo pathway component genes (unpublished observations), we examined mRNA levels of Hippo pathway components after induction of YAP activity. Notably, mRNA levels of *KIBRA*, *PTPN14* (protein tyrosine phosphatase, non-receptor type 14) and *AMOTL2*(angiomotin-like 2) showed expression patterns resembling those of *LATS2* and YAP target genes; in each case, mRNA levels increased transiently at the 2-hour time point following induction of wild-type YAP and were constitutively increased following induction of the YAP5SA mutant (Figure S2). Interestingly, KIBRA and PTPN14 cooperate to activate LATS [25–27] and AMOTL2 also can activate LATS [28, 29]. Judging from the changing patterns of mRNA levels, previously reported up-regulation of some other Hippo components, such as MST1 and AMOT, by the activity of YAP in several mouse models would be induced indirectly [23, 30, 31].

### TEAD is required for transactivation of LATS2 by YAP

**Figure 2:**
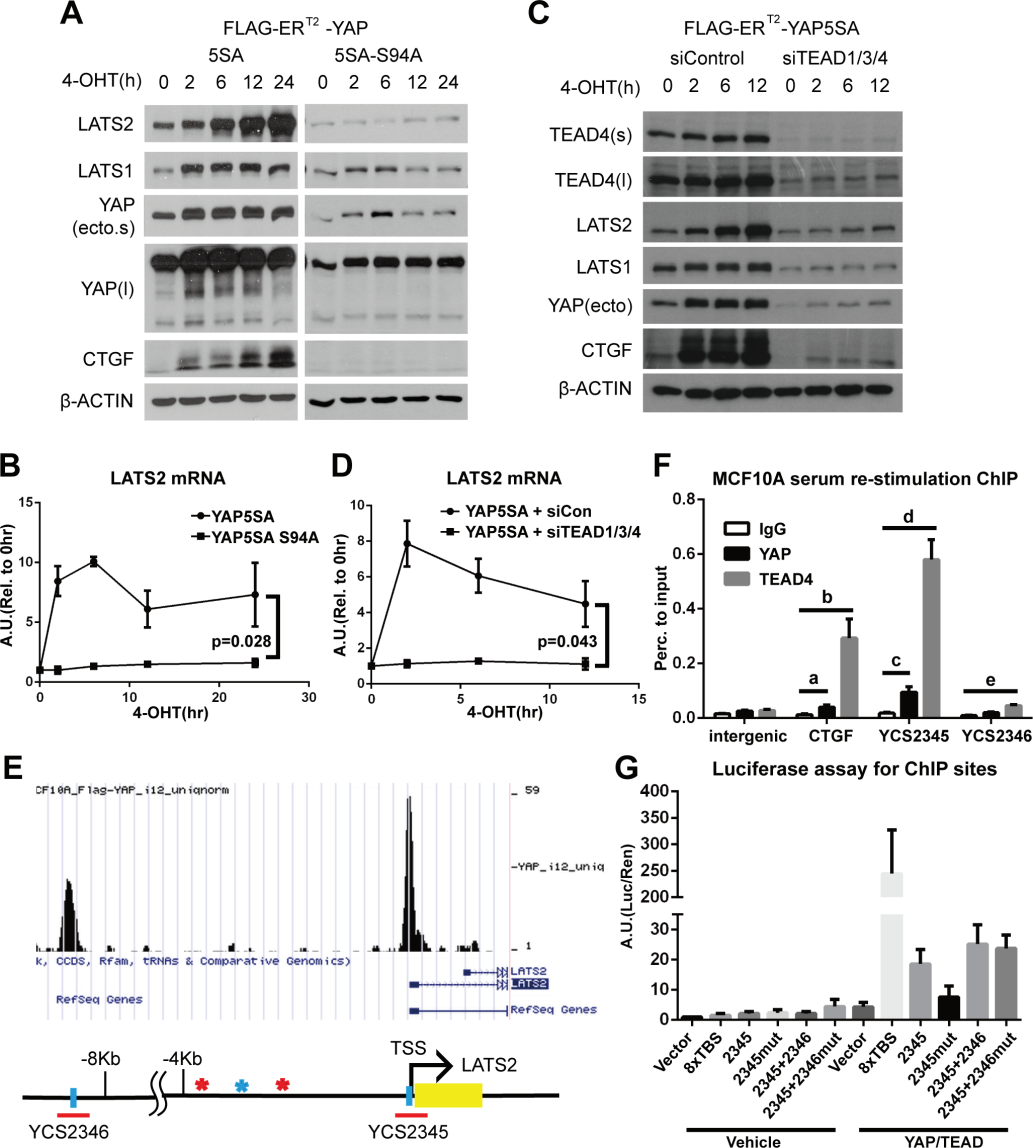
The YAP-TEAD complex directly increases LATS2 transcription (**A** and **B**) MCF-10A cells expressing YAP-5SA or YAP-5SA-S94A, which cannot bind to TEAD TFs, were treated with 4-OHT for the indicated times, and LATS2 levels were analyzed by Western blot (A) and qRT-PCR (B). *p*-value from ANOVA between two cell lines is indicated in panel (B). (**C** and **D**) YAP activity in YAP-5SA–expressing MCF-10A cells transfected with control or TEAD1/3/4 siRNA were induced with 4-OHT, and LATS2 protein (C) and mRNA (D) levels were determined. *p*-value from ANOVA between two experimental sets is indicated in panel (D). (**E**) The human *LATS2* promoter region. Yellow box indicates exons. Blue vertical bars indicate TEAD-binding motifs in each interval of ChIP-seq peaks (data not published), denoted by red horizontal bars; the corresponding peaks are illustrated using the UCSC genome browser (http://genome.ucsc.edu/). Colored asterisks indicate other TEAD-binding motifs in either orientation (red, CATTCC; blue, GGAATG). Red bar indicates regions primarily confirmed by ChIP-PCR analysis and subjected to further analysis. (**F**) ChIP assays for endogenous YAP and TEAD4 were performed using MCF-10A cells re-stimulated with serum/EGF after 24 hours of serum starvation. Enrichment of DNA fragments around TEAD-binding motifs in the LATS2 promoter was analyzed by qPCR. Binding was calculated as a percentage to input. *p*-value from two-tailed *t* test for each comparisons are as follows: a = 0.030, b = 0.016, c = 0.017, d = 0.0016 and e = 6.1 × 10^−4^. (**G**) Transcription-activating functions of TEAD-binding motifs in the *LATS2* promoter were evaluated by luciferase reporter assays. Mutated TEAD-binding motifs within an interval are indicated by ‘mut’ after each interval number. YCS2346 was evaluated in combination with YCS2345 since the interval is far from the TSS and thus could act as an enhancer. 8XTBS, eight tandem TEAD-binding sites (positive control).

We sought to determine which domain of YAP is responsible for inducing LATS2 expression by testing constitutively active YAP5SA harboring additional deletion or point mutation. Whereas YAP5SA lacking the proline-rich region at the N-terminal (ΔPRR) or WW domain (ΔWW) or containing a Y357F mutation still increased LATS2 protein levels, a TEAD binding-deficient mutant (YAP5SA-S94A) failed to increase LATS2 expression (Figure 2A and 2B, Figure S3A). These results suggest that YAP activates LATS2 transcription through the TEAD-binding motif. We next tested whether TEAD TFs are required for LATS2 induction by YAP. Depletion of TEAD TFs with siRNA blocked the YAP-mediated up-regulation of LATS2 transcription (Figure 2C and 2D). Consistently, luciferase assays using *LATS2* promoter region showed that co-expression of YAP and TEAD2 synergistically increased reporter signal intensity. Notably, this synergy was suppressed by expression of YAP-S94A mutant or by expression of TEAD2-R95K, which is a mutant form of TEAD2 that cannot bind to DNA (Figure 1D).

We next performed ChIP assays to determine whether the YAP/TEAD complex directly induces LATS2 transcription. MCF-10A cells that were serum/EGF-starved for 24 hours followed by 90 minutes of serum/EGF re-stimulation to activate endogenous YAP, were subjected to ChIP assays using an anti-YAP antibody and an anti-TEAD4 antibody. Binding of YAP and TEAD4 to selected regions of the *LATS2* promoter containing TEAD binding motifs was confirmed by qPCR (Figure 2E and 2F). Notably, one of these regions, designated YCS2345, include the transcription start site(TSS) of *LATS2* and the TEAD binding motif in the region lies at −10bp from the TSS of LATS2. These results suggest that the expression of *LATS2* is induced through direct transcriptional activation by the YAP/TEAD complex. To further confirm the necessity of TEAD-binding motifs for each YAP-binding site of the *LATS2* promoter, we performed luciferase assays using *LATS2* promoter constructs with or without a mutation in the TEAD-binding motifs (GGAATG → GGAGGG) (Figure 2G). Mutation of TEAD-binding motifs decreased the luciferase signal intensity, indicating that the TEAD-binding motif is required for LATS2 transcriptional induction by YAP. On the basis of these findings, we conclude that the YAP-TEAD complex binds to *LATS2* promoter to directly induce *LATS2* transcription.

### Deletion of LATS2 accelerates YAP-induced tumorigenesis in mouse liver

**Figure 3:**
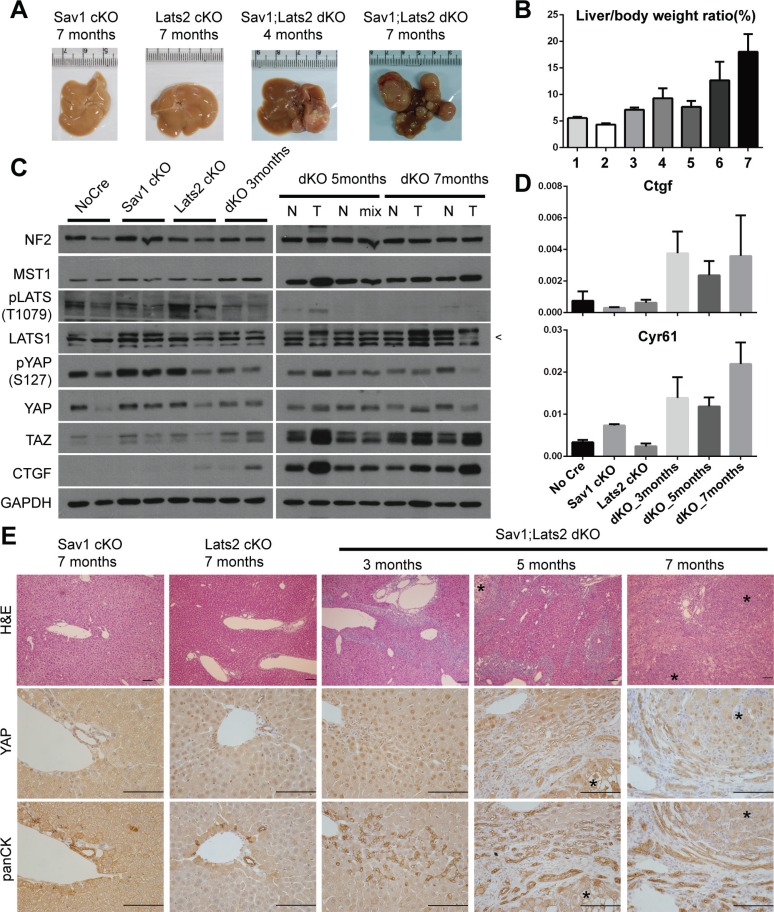
Ablation of negative feedback on YAP accelerates the YAP activity-induced mouse liver phenotype (**A**) Representative images of livers from mice with liver-specific knockout of the indicated genes at the indicated ages. (**B**) Weight ratio of the liver to the whole body of mice. Genotypes and ages of each bar are as follow; bar1-*Sav1 cKO* 7 months, bar2-*Lats2 cKO* 7 months, bar 3 to 7-*Sav1;Lats2 dKO* of 3, 4, 5, 6 and 7 months. (*n* ≥ 3 for each). (**C**) Hippo pathway components in liver tissues from each mouse of the indicated genotype and age were examined by Western blot analysis. N, normal tissue; T, tumor node; Mix, mix of normal and tumor tissue portions (necessitated by small node size). Pointer at right in LATS1 blot indicates the correct band. (**D**) mRNA levels of the YAP target genes *Ctgf* and *Cyr61* relative to *Gapdh* in liver tissues from the indicated genotypes and ages (*n* ≥ 3 for each). (**E**) Representative liver sections of mice with liver-specific knockout of the indicated genes at the indicated ages showing H & E staining and immunohistochemistry for YAP and cytokeratins. Asterisks in sections denote tumor nodes. Scale bars: 200 μm.

Although foregoing results illustrate the molecular-level mechanism implying negative feedback on YAP/TAZ activity in various cell lines, overexpression of YAP in these experiments could conceivably influence the outcome. Therefore, we attempted to confirm the existence of negative feedback regulation of YAP/TAZ activity and establish its physiological relevance using a mouse model in which the status of *Yap/Taz* is not changed. To this end, we took advantage of an existing liver-specific *Sav1*-knockout mouse model (*Sav1^**flox/flox**^; Albumin-Cre, Sav1-cKO*), which exhibits a tumorigenic phenotype that is derived from YAP [23]. *Sav1*-cKO mice develop spontaneous tumors after 1 year. If the negative feedback through LATS2 exists *in vivo*, abrogation of this negative feedback loop by *Lats2* deletion should accelerate tumorigenesis in a liver-specific *Sav1;Lats2* double-knockout mouse model (*Sav1^flox/flox^; Lats2^**flox/flox**^; Albumin-Cre, Sav1;Lats2-dKO*). As predicted, *Sav1;Lats2-dKO* mice exhibited liver tumors as early as 4 months, and tumors were fully developed within 7 months (Figure 3A). In contrast, liver-specific *Lats2* single-knockout mice (*Lats2*^flox/flox^; *Albumin-Cre*, *Lats2-cKO*) showed no overt histological abnormalities and did not develop liver tumors up to 16 months of age (data not shown). Liver/body weight ratios trended higher with age in *Sav1;Lats2-dKO* mice (Figure 3B). Consistent with predictions, Western blotting and qRT-PCR analyses showed increased YAP activity as the expression levels of its target genes such as Ctgf and Cyr61 were up-regulated in *Sav1;Lats2-dKO* mice (Figure 3C and 3D).

Hyperplasia of ductal/progenitor-like cells is a common phenotype of livers in which Hippo components (e.g. *Sav1, Mst1/2, Nf2*) are deleted [23, 32, 33]. Consistently, morphological analyses of H & E-stained sections of *Sav1;Lats2-dKO* livers showed hyperplasia of ductal/progenitor-like cell populations that have a high nuclear/cytoplasmic ratio (Figure 3E). These cells indeed showed specific expression of cytokeratins and A6, which are known markers for liver progenitor cells (Figure 3E, Figure S4). Expression and nuclear localization of YAP were notable in sections from *Sav1;Lats2*-*dKO* mice compared with those from *Sav1-cKO* or *Lats2-cKO* mice. The staining pattern and morphology of cells appeared to be similar to that of previously reported YAP-induced hyperplastic regions and tumors (Figure 3E) [23, 34]. Collectively, these results suggest the presence of a negative feedback mechanism in mouse liver that regulates YAP through LATS2 and exerts a tumor-suppressive function.

### Abrogation of negative feedback on YAP induces a tumor-associated phenotype in cell lines

**Figure 4:**
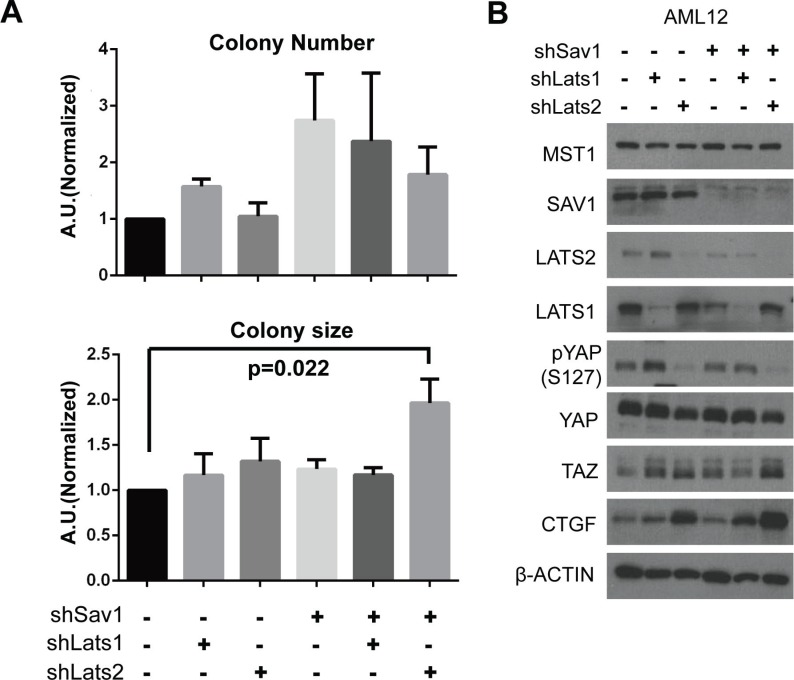
Abrogation of negative feedback on YAP in a cell line causes a tumor-associated phenotype (**A**) Colony formation in soft agar was assessed in AML-12 cell lines with the indicated modifications. *p*-value from two-tailed *t*-test between results of control cells and *Sav1;Lats2* double-knockdown cells is indicated. (**B**) Molecular changes in core Hippo components caused by *Sav1* knockdown and subsequent *Lats* knockdown. The same cells used in panel (A) were plated and harvested at 90% confluence.

To recapitulate the tumorigenic phenotype shown in the mouse model in which negative feedback on YAP/TAZ is ablated, we characterized tumor-associated cellular phenotypes in the AML-12 normal mouse liver cell line after depletion of SAV1, LATS1, and/or LATS2 proteins with shRNA constructs. Colony forming assays revealed significant differences in growth behavior in soft agar among variously transduced cells. *Sav1; Lats2* double-knockdown specifically increased the size of colonies, without notably affecting colony numbers (Figure 4A). These results suggest that LATS2 depletion boosts tumorigenic effects of SAV1 depletion in cells. We next examined molecular changes in core Hippo components in *Sav1; Lats2* double-knockdown cells by Western blot analysis. The decreased ratio of phospho-YAP to total YAP protein, increased TAZ level, and increased expression of CTGF indicate that the inhibitory power on YAP/TAZ by the Hippo pathway is substantially diminished by double-knockdown of SAV1 and LATS2 (Figure 4B). With these results, we suggest that the accelerated tumorigenesis observed in the *Sav1; Lats2-dKO* mouse model originates from the failure of YAP/TAZ activity suppression owing to abrogation of the negative feedback mechanism in the Hippo pathway.

### Negative feedback in the Hippo pathway is conserved in drosophila

**Figure 5:**
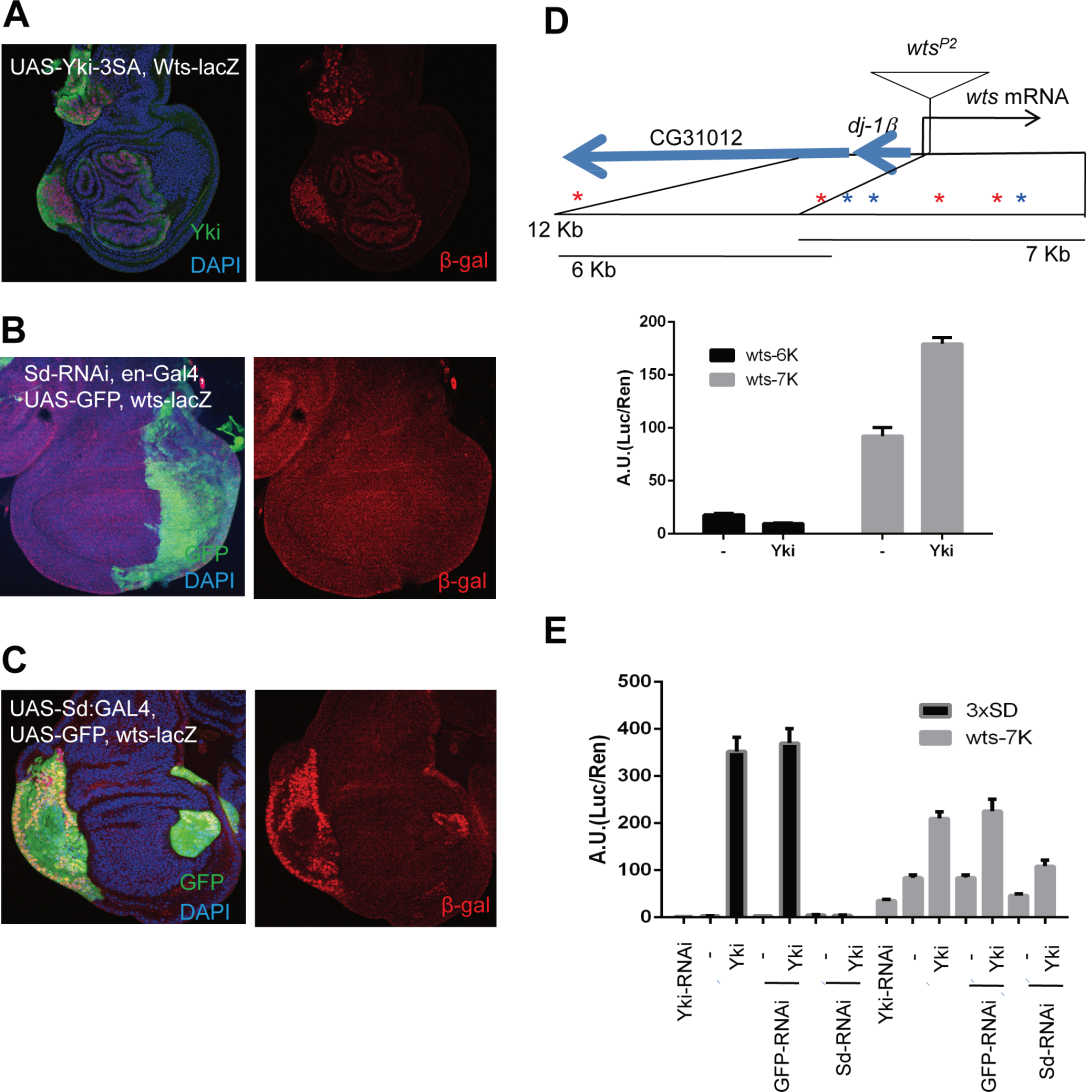
Direct negative feedback on YAP/Yorkie is conserved in *Drosophila* (**A**) Wing discs with *wts-lacZ* (*wts^P2^*) stained with β-gal (red). *Ay-Gal4* flip-out clones of UAS-Yki-3SA are marked by overexpressed Yki. Pictures are composites of multiple confocal sections. (**B**) Wing discs expressing UAS-Sd RNAi at the posterior region using *en-Gal4*, marked by UAS-GFP. *wts-lacZ* was monitored by β-gal staining. (**C**) Wing discs expressing UAS-Sd:GA using Ay-Gal4, where the flip-out clones were marked by UAS-GFP, and wts-lacZ was monitored by β-gal staining. Nuclei were stained with DAPI. (**D**) Promoter region of *wts* showing transcription start, insertion of *wts^P2^* and translation start sites. Seven conserved Sd-binding sites, three forward (blue stars) and four reverse (red stars), are present within the 12-kb promoter region. Expression driven by the promoter region was analyzed by luciferase assays in S2 cells. Histograms indicate average ratios of firefly luciferase (experimental)/Renilla luciferase (control) from triplicate experiments. Error bars indicate standard deviation. (**E**) Effects of Yki-RNAi, GFP-RNAi (negative control), and Sd-RNAi on Yki responsiveness determined by luciferase reporter assays in S2 cells using the luciferase constructs with 3xSd-binding motif or *Wts*-7K promoter region.

Since the Hippo pathway is well conserved in Drosophila and mammals, we investigated whether the negative feedback loop demonstrated above also exists in Drosophila using *wts^P2^*, a lacZ enhancer trap insertion within the *Warts* locus (Figure 5D). We found that *wts^P2^* β-gal expression was up-regulated in flip-out clones expressing active Yorkie (Figure 5A) compared to the barely detectable *wts^P2^* β-gal signal in wing discs under normal conditions. To determine if the regulation of Warts by Yorkie requires Scalloped, in the same manner as mammalian YAP needs TEADs to induce LATS2, we examined *wts-lacZ* (*wts^P2^*) expression following RNAi-mediated depletion of Scalloped in the posterior part of wing disc using *en-Gal4* to drive expression of a UAS-sd shRNA. As expected, expression of *wts-lacZ* was down-regulated in Scalloped-depleted regions, although the magnitude of this change appeared modest owing to low β-gal signal intensities (Figure 5B). Conversely, *wts-lacZ* was up-regulated in flip-out clones overexpressing Sd:GA (Figure 5C), an activated form of Scalloped in which the Gal4 activation domain is fused to full-length Scalloped [35]. The role of Yorkie and Scalloped in *Warts* expression was further assessed using luciferase reporter assays in S2 cells expressing a 7 kb sub-fragment of the *Warts* promoter through which Yorkie induces luciferase expression (Figure 5D). Luciferase signals were determined following expression and deletion of Yorkie and Scalloped, respectively. Consistent with wing disc results, knocking down Scalloped significantly impaired activation of the *Warts* promoter-reporter by Yorkie. These results thus indicate that a simple and direct form of negative feedback in the Hippo pathway is evolutionarily conserved in Drosophila as Yorkie induces Warts transcription, just as YAP induces LATS2 transcription in mammalian systems.

### Specific induction of LATS2 than LATS1 by YAP reflects their functional difference

While protein levels of LATS2 is significantly up-regulated and accumulated according to YAP/TAZ activity, protein levels of LATS1 did not show such correlation to YAP/TAZ activity although ectopic expression of YAP and its mutants increased LATS1 protein in MCF 10A cells (Figures 1A, 2A and S3A). However, we observed that transcription of *LATS1* was not induced by YAP/TAZ activation (Figure S5A and S5B). To investigate differential role of LATS1 and LATS2 in the negative feedback context, we knocked-down each paralog and induced YAP activity. Disappointedly, both single knockdown of *LATS1* or *LATS2* did not hinder the negative feedback phenomenon (Figure S5C and S5D). This result implicates that LATS1 and LATS2 participate in the negative feedback of the Hippo pathway. However, we speculated that there would be a functional difference between two paralogs in the context of the negative feedback since only LATS2 is induced by YAP. To demonstrate such difference, we investigated liver sections of liver-specific *Sav1;Lats1* double-knockout mouse model(*Sav1^flox/flox^*; *Lats1^flox/flox^*; *Albumin-Cre*, *Sav1;Lats1-dKO*). Interestingly, the degree of hyperplasia and invasion of ductal/progenitor-like cells in the *Sav1;Lats1*-*dKO* mice was much less than that of *Sav1;Lats2-dKO* mice (Figure S6A and Figure 3E). Additional deletion of one *Lats2* allele, so that the only one *Lats* allele is remained, result in more progressed phenotype. However, the degree of hyperplasia and invasion of ductal/progenitor-like cells shown in livers from 6 months old mice with genotype of *Sav1^flox/flox^; Lats1^flox/flox^; Lats2^flox/^+; Albumin-Cre* was only comparable or less than that of 3 months old *Sav1;Lats2-dKO* mouse livers which still have two *Lats1* alleles (Figure S6A and Figure 3E). Increasing YAP activity by deletion of *Lats1* and *Lats2* alleles was confirmed by Western blot and qRT-PCR showing a tendency of decreasing pYAP/YAP ratio and increasing expression of YAP target genes such as Ctgf and Cyr61 (Figure S6B and S6C). These results suggest that LATS2 is more important than LATS1 in the context of tumor suppression at least in the liver through the negative feedback of the Hippo pathway.

## DISCUSSION

Functionally, the Hippo pathway is a tumor-suppressive pathway that represses YAP/TAZ oncoproteins. Canonical Hippo pathway, named from its historical relevance, functions through MST1/2 and the core kinase cassette. Additionally, some signaling cues can activate LATS1/2 independent of MST1/2. For example, G protein-coupled receptors (GPCRs) can activate or repress LATS1/2, presumably though the Rho-actin axis [18]. Actin filament formation represses LATS activity, whereas disruption of the actin cytoskeleton through detachment of cells or drug treatment activates LATS kinases, thereby down-regulating YAP/TAZ activity [14, 19, 36, 37]. Interestingly, restrictions on the growth area of a cell or reduction of cytoskeletal tension from the surrounding matrix may repress YAP/TAZ activity directly [13, 38]. Finally, AMOT (angiomotin) and AMOTL1/2 can bind and retain YAP/TAZ in the cytoplasm regardless of their phosphorylation status [39–42].

In addition to aforementioned variety of upstream cues, here we show the negative feedback regulation of YAP/TAZ activity. YAP/TAZ induce transcription of some Hippo pathway components, among which LATS2 is the most prominent target gene investigated. We further showed that TEAD TFs complex with YAP and directly bind to the *LATS2* promoter region. YAP-induced liver tumorigenesis in *Sav1*-knockout mice was accelerated by concurrent deletion of *Lats2*. Moreover, such synergistic enhancement of tumorigenesis was not observed when *Lats1* was additionally deleted. A similar phenotype was also observed in *Sav1; Lats2* double-knockdown cells, which formed larger colonies in soft agar. Notably, we also found that this negative feedback loop is conserved in *Drosophila*, as evidenced by the induction of Warts transcription by the Yorkie-Scalloped complex.

Recently, two papers provided mechanisms for homeostatic control of YAP/TAZ in the Hippo pathway [30, 31] while we were preparing this manuscript. In particular, it was reported that YAP induces LATS2 transcription and stimulates the kinase activity of LATS1/2 though NF2 [31]. Our study also indicates that transactivation of LATS2 is at the center of the negative feedback mechanism. Importantly, we confirmed that this mechanism is evolutionarily conserved. Moreover, we were able to distinguish the role of LATS1 and LATS2 in the context of the negative feedback using mouse models. We noted that induction of NF2 expression by YAP activation is cell-context dependent (Figure S1) and its mRNA expression pattern after YAP activation was not correlated with that of known YAP target genes and some Hippo upstream components; *AMOTL2*, *KIBRA* and *PTPN14* (Figure S2). These results may reflect the complicated role of NF2 in the Hippo pathway which involve EGFR, AMOT and the Ras-MAPK pathway [32, 43, 44]. Nevertheless, LATS kinases are essential for homeotic regulation of YAP/TAZ, and thus how a variety of Hippo upstream cues and components control LATS activity should be rigorously investigated in the future.

Interestingly, transcription of *LATS2*, but not *LATS1*, is specifically induced by YAP/TAZ activity. However, knock-down of any single paralog of LATS did not ablate the negative feedback phenomenon (Figure S5C and S5D). This confounding situation can be interpreted in light of the fact that YAP induces expression of other upstream components of the Hippo pathway. A slight initial up-regulation of Hippo upstream components and subsequent activation of existing LATS kinases would confer sufficient regulatory power on YAP. However, if YAP activity is sustained and ultimately escapes this primary regulation, LATS2 would act as a back-up system to further repress YAP/TAZ activity. This idea is supported by our observations that colony size in soft agar assays was specifically larger for *Sav1; Lats2* double-knockdown cell lines (Figure 4) and that the YAP related phenotype from *Sav1;Lats2-dKO* mice was much severe than that from *Sav1;Lats1-dKO* mice (Figures 3 and S6).

Interestingly, we observed an increment of LATS1 protein by ectopic expression of YAP constructs (Figures 1A, 2A and S3A). This seems to be dependent on TEAD (Figure 2A and 2C). Since LATS1 is not a target of YAP and actinomycin D treatment failed to inhibit the induction (Figure 1C), translational or post-translational mechanisms would be the cause. One possible explanation is that LATS1 could be stabilized by upstream Hippo components induced by YAP/TEAD complex. In addition to this hypothesis, other mechanisms by which LATS1 expression is regulated would be an important question.

The hypothesized functional differences between LATS1 and LATS2 in the negative feedback on YAP/TAZ, noted above, may reflect differences in kinetics and dynamics. For example, a single-cell level analysis of the dynamics of the p53-MDM2 negative feedback loop revealed essentially digital behavior [45]. In the case of NF-κB and IκB, only one of the three isoforms of IκB is a target of NF-κB, similar to LATS1 and LATS2. A modeling study of NF-κB-IκB negative feedback loop kinetics showed that each IkB isoform differentially controlled NF-κB [46]. As illustrated in the above two studies, understanding the kinetics and dynamics of the negative feedback of the Hippo pathway will be important to know how and to what extent YAP/TAZ activity is regulated.

## MATERIALS AND METHODS

### Plasmids

Viral vectors for expression of inducible YAP and its mutants were created in the vectors pMSCV puro or pMSCV hygro. FLAG tag sequence was introduced with BglII/BamHI and YAP constructs were inserted with BamHI/EcoRI. Then, a construct for modified estrogen receptor ligand binding domain sensitive to 4-OHT(ER^T2^) was introduced with BamHI in both ends.

### Cell culture

MCF-10A human mammary epithelial cells were cultured in DMEM/F12 media supplemented with epidermal growth factor (EGF, 20 ng/ml), hydrocortisone (0.5 μg/ml), cholera toxin (100 ng/ml), insulin (10 μg/ml) and horse serum (5%). NMuMG (normal murine mammary gland), HaCaT (human keratinocyte) and AML-12 (normal mouse liver) cell lines were cultured according to American Type Culture Collection (www.atcc.org). Transfections were performed with polyethylenimine. Lipofectamine RNAiMAX Reagent (Life Technologies) was used for transfection of small interfering RNA (siRNA). siRNA sequences for TEAD1/3/4, LATS1 and LATS2 are as previously described [8, 13].

### Western blot

Western blot analyses were performed using a standard protocol. Briefly, RIPA buffer (50 mM Tris-Cl pH 7.5, 150 mM NaCl, 1 mM EDTA, 1% NP-40, 0.5% deoxycholate, 0.1% SDS) containing protease inhibitors and phosphatase inhibitors (Pepstatin A 1 μg/ml, Leupeptin 1 μg/ml, 1 mM Phenylmethylsulfonyl Fluoride, 1 mM Sodium Orthovanadate, 5 mM Sodium Fluoride) was used for lysis of cell pellets and homogenization and lysis of mouse liver tissues. Supplementary Text antibodies used.

### Luciferase assay

Luciferase constructs and each indicated DNA construct were co-transfected with a Renilla luciferase construct, used as a control for transfection efficiency. Luciferase assays were performed using the Dual-Luciferase Reporter Assay System (Promega) following the manufacturer's guide. Luciferase signal intensities were calculated relative to those of Renilla luciferase.

### Quantitative polymerase chain reaction

RNA preparation and cDNA synthesis were done as described by the manufacturer using RiboEx (GeneAll) and M-MLV reverse transcriptase (Enzynomics). Quantitative polymerase chain reaction (qPCR) was performed using a SYBR green premix reagent (TOPreal qPCR 2X PreMIX; Enzynomics) and Bio-Rad CFX Connect instrument. Results were analyzed using Microsoft Excel.

### Chromatin immunoprecipitation

Cells were washed once with phosphate-buffered saline (PBS) and fixed in 1% formaldehyde in PBS for 20 minutes at room temperature. Formalin was quenched with 125 mM glycine, after which cells were harvested. Following procedures were described in Supplementary Text. The anti-YAP antibody and anti-TEAD4 antibody used for Western blotting was also used for immunoprecipitating genomic DNA fragments, which were analyzed by qPCR. qPCR results were analyzed using Microsoft Excel.

### Mice

All mice used in this research were bred and maintained under specific pathogen-free conditions according to guidelines of the Korea Advanced Institute for Science and Technology. Liver-specific *Sav1*-knockout mouse model and *Lats2*^flox/flox^ mouse line were generated as previously described [14, 23]. Lats1 ^lox/flox^ mouse was generated and gifted by Randy L. Johnson (M.D. Anderson Cancer Center).

### Immunohistochemistry

Paraffin-embedded tissues were cut into 4-μm-thick sections and used for hematoxylin and eosin (H & E) staining or immunohistochemistry. YAP was immunostained using the same antibody as that used for Western blotting, and cytokeratin was immunostained with a pan (wide-spectrum) antibody (DAKO). Immunoreactive proteins were detected with a horseradish peroxidase (HRP)-conjugated anti-rabbit secondary antibody (Jackson ImmunoResearch) and visualized using a DAB substrate kit (Vector Laboratories).

Immunohistochemistry procedures for frozen sections (10 μm) were similar to those used for paraffin-embedded sections. Anti-A6 (kindly provided by V. Factor) and pan-cytokeratin antibodies were detected using the DAB substrate kit and Vectostain Elite ABC Kit (Vector Laboratories), respectively.

### Soft agar assay

Cell culture grade agar (Sigma) was dissolved in sterile water using a microwave. Bottom agar in media (0.5% agar) was plated in the wells of a 6-well plate. Trypsinized cells (5000 cells per well) were then suspended in pre-warmed top agar in media (0.34% agar), then plated atop the bottom agar. Medium (3 ml per well) was added the next day. Plates were incubated for 2 weeks and media were changed every 3 days. Colonies were stained with crystal violet. Images were acquired for one random region per well. The number and size of colonies were analyzed using the ImageJ program.

### Statistical analysis

Analysis of variance (ANOVA) and two-tailed *t*-tests were used for statistical analyses. All analyses were performed using Prism6 (GraphPad Software). Error bars indicate S.E.M. unless otherwise specified.

## SUPPLEMENTARY MATERIALS FIGURES


