# Supplementary material for: An evolutionarily conserved negative feedback mechanism in the Hippo pathway reflects functional difference between LATS1 and LATS2
Source: Oncotarget. 2016 Mar 19;7(17):24063–75. doi: 10.18632/oncotarget.8211 (PMC5029684; doi:10.18632/oncotarget.8211)
Supplement: Supplementary file 1 [file oncotarget-07-24063-s001.pdf]

An evolutionarily conserved negative feedback mechanism in the hippo pathway reflects functional difference between LATS1 and LATS2

Supplementary Materials

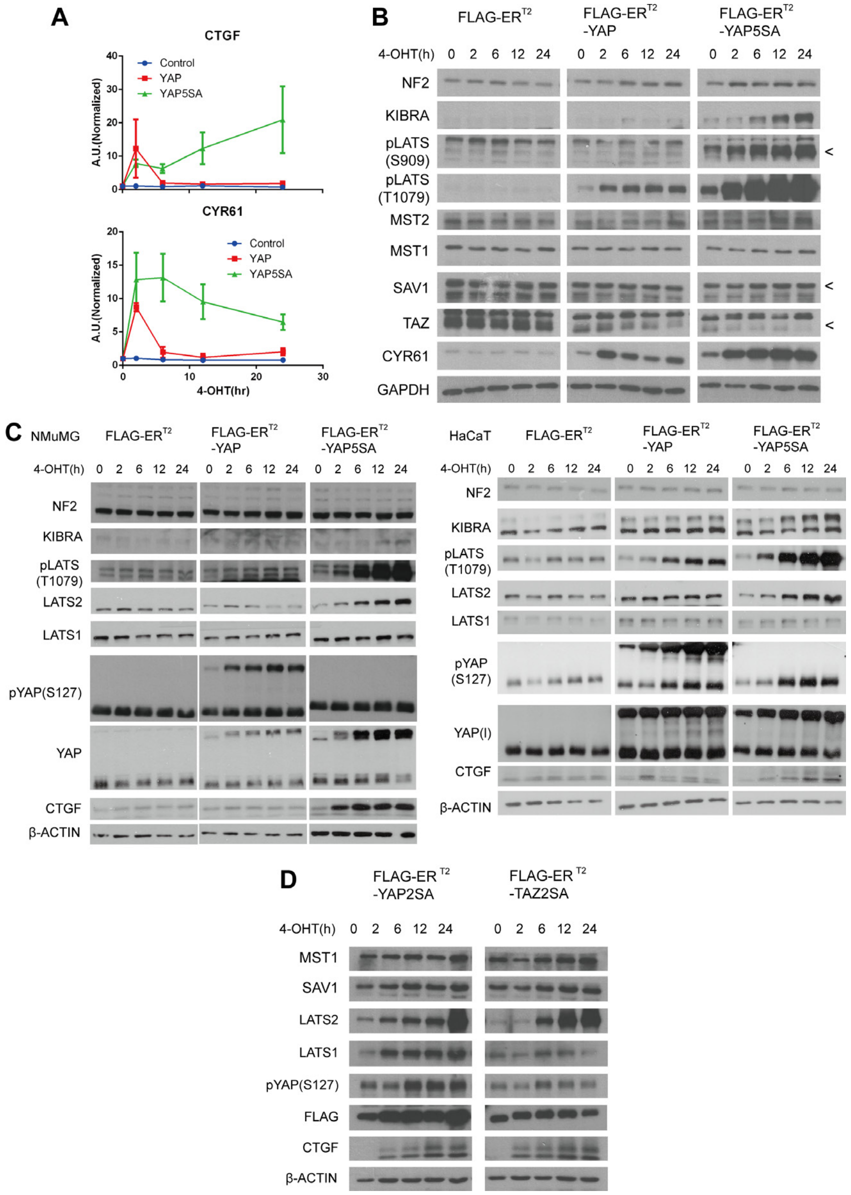

Supplementary Figure S1: Effects of YAP activation on other Hippo pathway components and in other cell lines. (**A**) mRNA levels of CTGF and CYR61 were measured by qRT-PCR for the same cell line set used in Figure 1A. YAP target genes were transiently induced at 2 hours by wild-type YAP and constitutively up-regulated by the YAP5SA mutant. Results were normalized to 0-hour controls for each cell line. (**B**) Hippo pathway components other than LATS kinases and LATS phosphorylation status were assessed in MCF-10A cells used in Figure 1A by Western blot analysis after induction of YAP activity using 4-OHT. MST1/2 and SAV1 protein levels were not significantly changed. Phospho-LATS levels were correlated with total LATS protein level, indicating that the increase in active LATS kinase level is mainly attributable to an increase in LATS proteins. NF2 was slightly increased and KIBRA expression was also increased. Pointers at the right side of blots indicate the specific band for each protein. (**C**) Up-regulation of LATS2 upon YAP activation was consistently observed in NMuMG and HaCaT cell lines. KIBRA protein was also increased, whereas NF2 was unchanged. (**D**) Induction of LATS2 by TAZ activation was assessed by Western blot analysis in MCF-10A cells expressing the indicated constructs after treatment with 4-OHT for up to 24 hours. LATS2 protein expression was dramatically increased in both YAP2SA- and TAZ2SA-expressing cells.

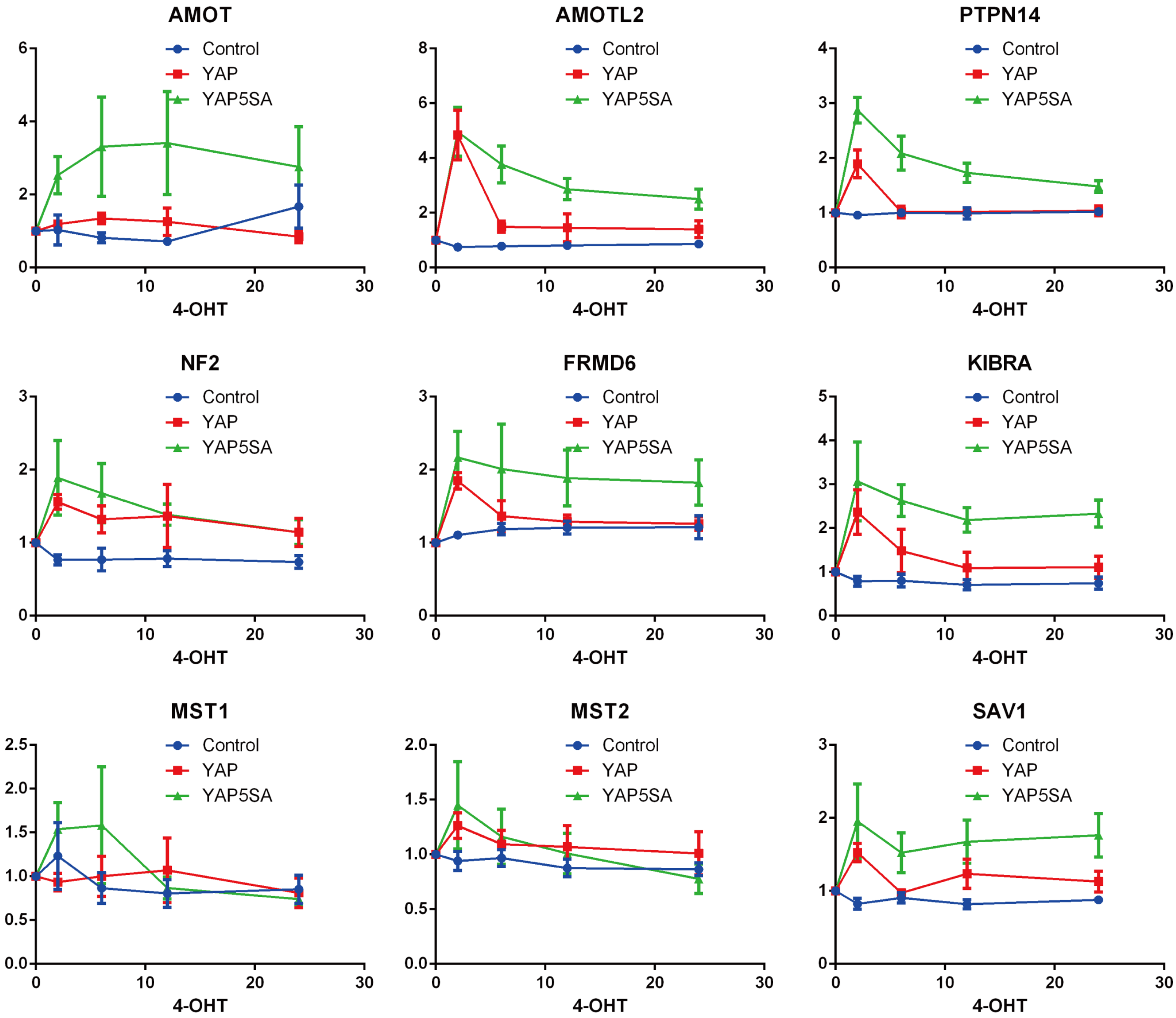

Supplementary Figure S2: Changes in mRNA levels of selected Hippo upstream components after induction of YAP activity. mRNA levels of each gene were analyzed by qRT-PCR from the same cell line set used in Figure 1A. The patterns of a transient peak at 2 hours in wild-type YAP-induced cells and continuously up-regulated expression in YAP5SA mutant-induced cells were easily noticeable for AMOTL2, PTPN14, and KIBRA. The patterns of SAV1 and FRMD6(Expanded ortholog [2, 3]) induction were similar, but expression levels were relatively low. Increased levels of AMOT mRNA in YAP5SA mutant-induced cells were observed but overall patterns among three cell lines are not fit to other YAP target genes. Expression of MST1/2 was largely unchanged.

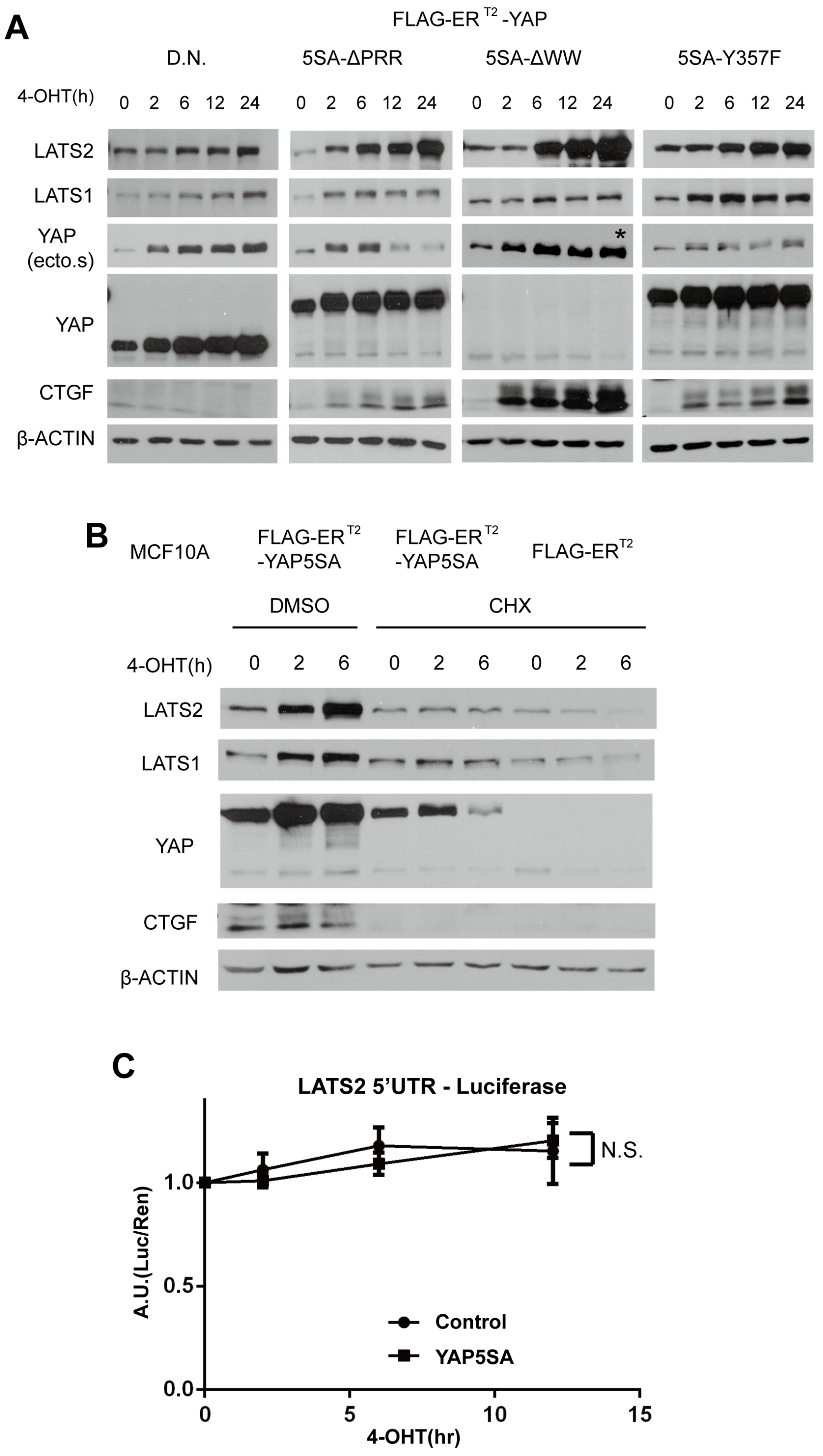

Supplementary Figure S3: Up-regulation of LATS2 by YAP activation mainly reflects transcriptional activation. (**A**) LATS2 protein levels were assessed following 4-OHT induction of YAP proteins with the indicated mutations; D.N., dominant-negative form of YAP lacking a C-terminal transactivation domain; ΔPRR, YAP lacking the N-terminal proline-rich region; ΔWW, YAP lacking two WW domains. Y357F, YAP substitution mutant containing a non-phosphorylatable phenylalanine in place of tyrosine 357, the target phosphorylation site of c-Abl that mediates YAP stabilization [4]. Ectopic YAP5SA-ΔWW protein was detected with an anti-FLAG antibody (asterisk). (**B**) LATS2 protein stability upon YAP activation was examined after cycloheximide (CHX) treatment. MCF-10A cells expressing the indicated construct were pre-treated with CHX 30 minutes before 4-OHT induction and then harvested at the indicated times. The slight apparent increase in the stability of both LATS1 and LATS2 caused by ectopic expression of YAP5SA is insufficient to account for the remarkable induction of LATS2 protein. (**C**) Effect of YAP activation on LATS2 translation was assessed using a luciferase reporter linked to the 5′-untranslated region (5′-UTR) of LATS2. LATS2 5′-UTR-luciferase expression was driven by a CMV promoter in NIH-3T3 cell lines bearing the indicated constructs. No notable changes in luciferase signal were observed.

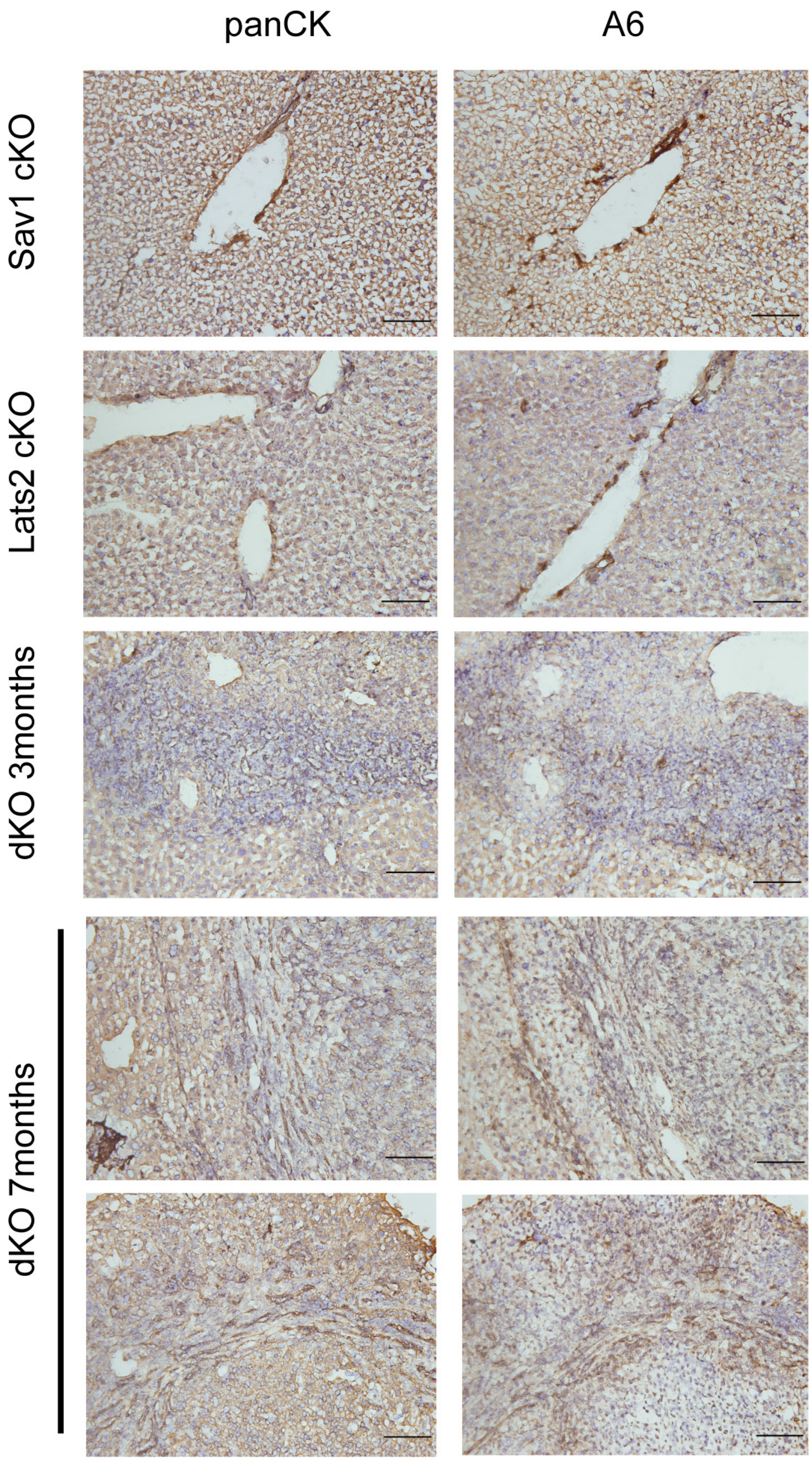

Supplementary Figure S4: Analysis of ductal/progenitor cell markers in hyperplastic liver cells from *Sav1;Lats2*-dKO mice. Cryosections of livers from mice of the indicated genotype and age were immunostained with anti-A6 and pan-cytokeratin antibodies. Similar patterns of expression of both markers were observed. Scale bars: 200 μm.

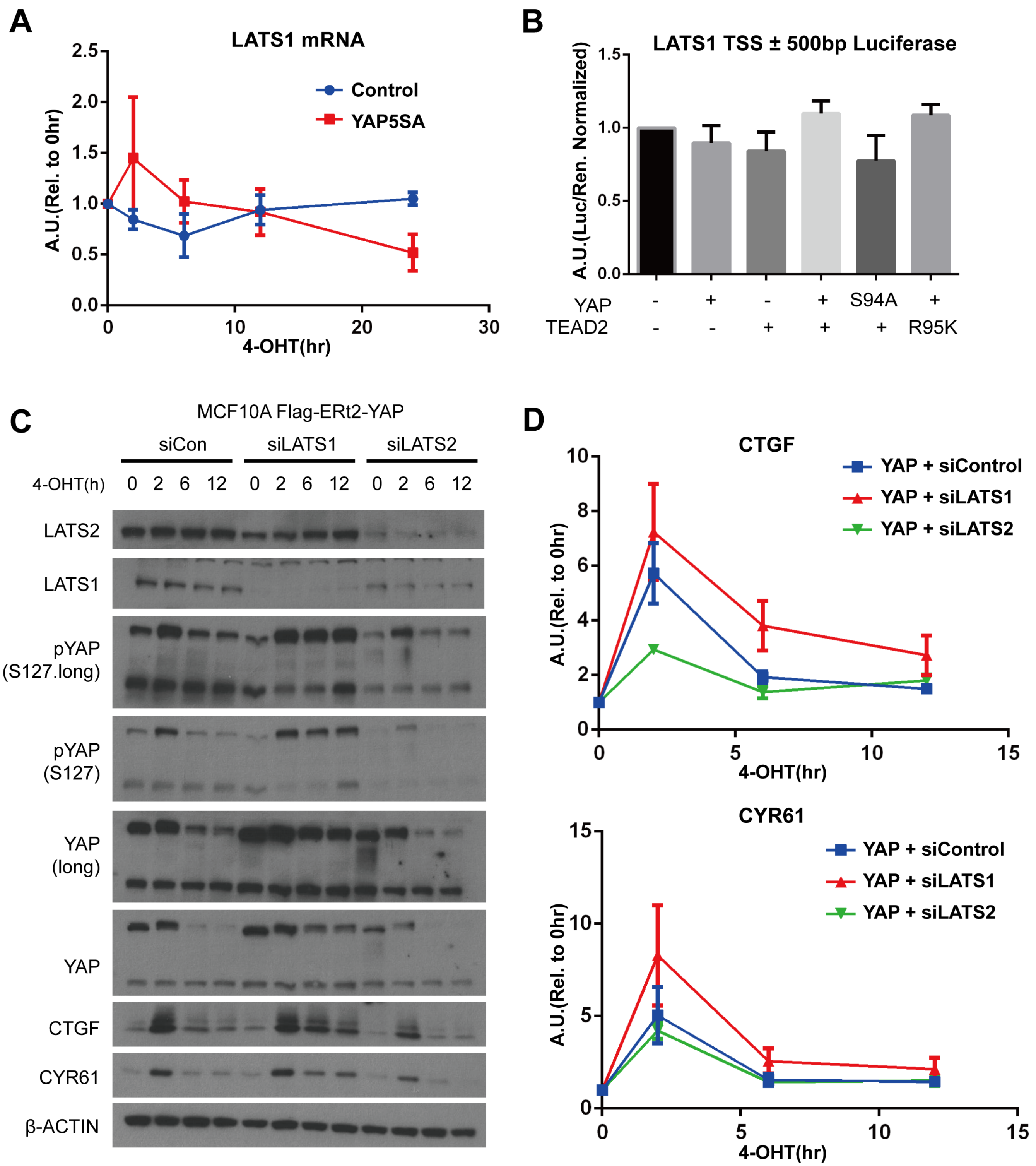

Supplementary Figure S5: LATS1 is not upregulated by YAP but can conduct the negative feedback mechanism. (**A**) MCF-10A cells transduced with control vector or inducible YAP5SA were treated with 4-OHT for up to 24 hours. LATS1 mRNA levels were quantified using qRT-PCR. The results show that LATS1 transcription was not specifically induced by YAP activity. (**B**) Luciferase reporter assays were performed using the promoter region (TSS+–500bp) of *LATS1*. HEK-293T cells were co-transfected with the indicated YAP/TEAD constructs and luciferase constructs, then luciferase signals were measured after 24 hours. (**C** and **D**) Effects of single knockdown of LATS1 or LATS2 on the negative feedback phenomenon in MCF-10A cells expressing inducible wild-type YAP. Changes in LATS kinases, YAP phosphorylation status, and YAP target genes were analyzed by Western blotting (C). qRT-PCR revealed a transient increase in CTGF and CYR61 mRNA (D). Overall, the feedback phenomenon was not abolished by knockdown of any single LATS paralog (see Discussion for details).

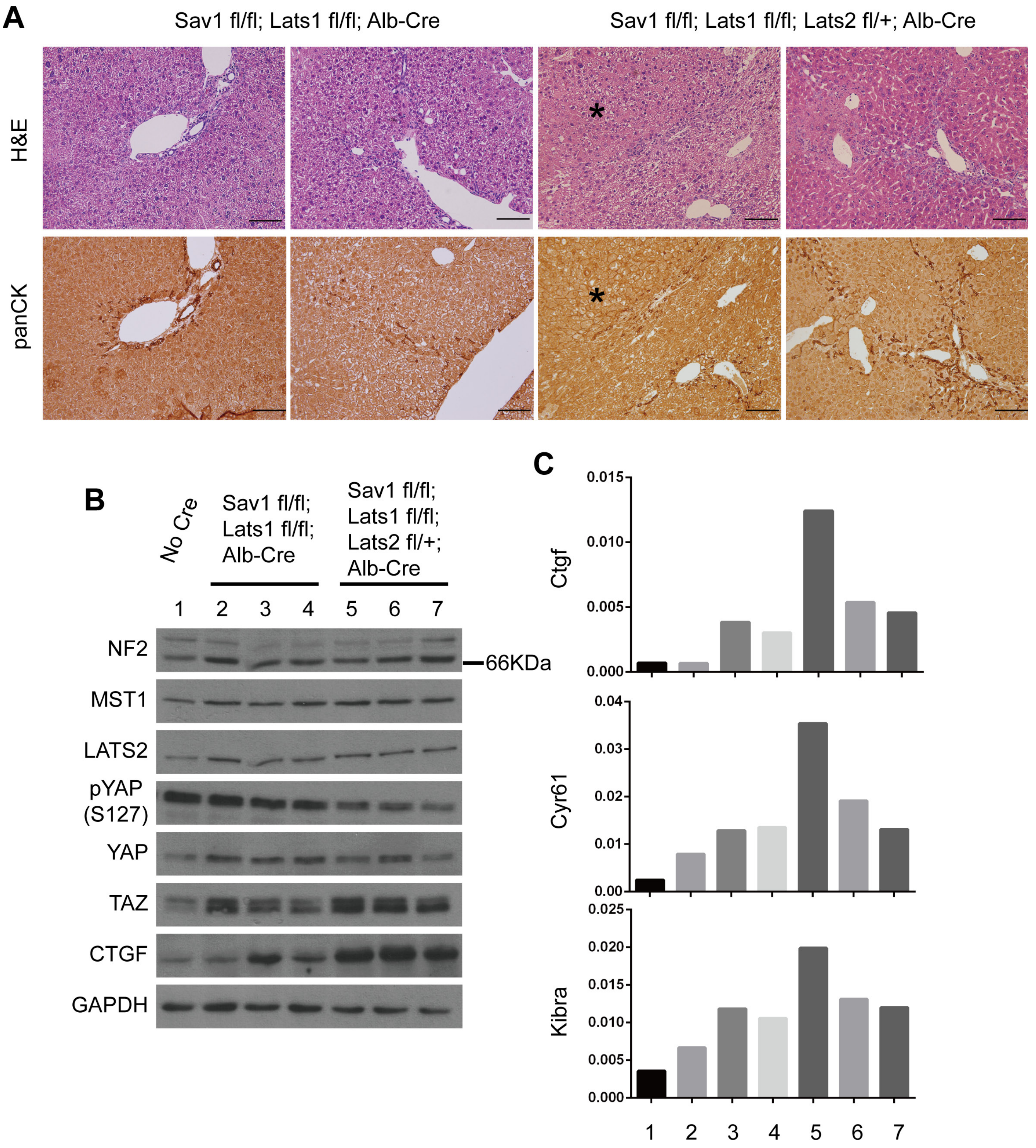

Supplementary Figure S6: Deletion of *Lats2* affects more than *Lats1* for YAP induced tumorigenesis. (**A**) Liver sections from mice with indicated genotype of 6 months age were subjected to H & E staining and immunohistochemistry for cytokeratins. Asterisks in sections denote tumor nodes. Scale bars: 200 μm. (**B** and **C**) Hippo pathway components of liver tissues from mice with indicated genotype of 6 months age were examined by Western blot analysis (B) and qRT-PCR (C). Each samples were distinguished by numbers. Trend of increasing YAP/TAZ activity by decreasing number of *Lats* allele is observable.

MATERIALS AND METHODS

Antibodies

Antibodies against the following proteins were used for Western blot: LATS2, S127-phosphorylated YAP (pYAP-S127), pLATS-T1079, pLATS-S909, MST1, MST2, KIBRA and TAZ, from Cell Signaling; GAPDH and TEAD4, from Abcam; β-actin, from Sigma; FLAG, from Wako; CTGF (connective tissue growth factor), CYR61 (cysteine-rich angiogenic inducer 6) and NF2, from Santa Cruz Biotechnology; and LATS1, from Bethyl Laboratories. Antibodies against YAP, SAV1, and pYAP-S381 were generated as previously described [1].

Primers

The following primer pairs designed to amplify the indicated targets were used for qRT-PCR: human β-actin, 5′-CAT GTA CGT TGC TAT CCA GGC-3′ (forward) and 5′-CTC CTT AAT GTC ACG CAC GAT-3′ (reverse); human LATS1, 5′-AAT TTG GGA CGC ATC ATA AAG CC-3′ (forward) and 5′-TCG TCG AGG ATC TTG GTA ACT C-3′ (reverse); human LATS2, 5′-ACC CCA AAG TTC GGA CCT TAT-3′ (forward) and 5′-CAT TTG CCG GTT CAC TTC TGC-3′ (reverse); human CTGF, 5′-ACC GAC TGG AAG ACA CGT TTG-3′ (forward) and 5′-CCA GGT CAG CTT CGC AAG G-3′ (reverse); human CYR61, 5′-CTC GCC TTA GTC GTC ACC C-3′ (forward) and 5′-CGC CGA AGT TGC ATT CCA G-3′ (reverse); human AMOT, 5′-AGG GCG AGA TTC GGA GGA T-3′ (forward) and 5′-CCT CTG ACC CCT CAT ATT CCT T-3′ (reverse); human AMOTL2, 5′-ACC ATG CGG AAC AAG ATG GAC-3′ (forward) and 5′-GGC GGC GAT TTG CAG ATT C-3′ (reverse); human PTPN14, 5′-GGG GGC CAT GTA AGC AG-3′ (forward) and 5′-GCA GTT TCG TGA CTG GAA AA-3′ (reverse); human NF2, 5′-CCA GCT ATG TAT CGG GAA CC-3′ (forward) and 5′-GCT CCA TCT GCT TTC TAG CC-3′ (reverse); human FRMD6, 5′-CCA CCT CTT TGG ACT CAG TGT-3′ (forward) and 5′-CAA ATT GGT CGA TAC CCT TGC T-3′ (reverse); human KIBRA, 5′-CTC CGA GGC CAG AGC TGT AAG GAA C-3′ (forward) and 5′-CTA GAG GAC TTG TGA CTC AGT AC-3′ (reverse); human MST1, 5′-CCT CCC ACA TTC CGA AAA CCA-3′ (forward) and 5′-GCA CTC CTG ACA AAT GGG TG-3′ (reverse); human MST2, 5′-CGA TGT TGG AAT CCG ACT TGG-3′ (forward) and 5′-GTC TTT GTA CTT GTG GTG AGG TT-3′ (reverse); human SAV1, 5′-ATG CTG TCC CGA AAG AAA ACC-3′ (forward) and 5′-AGG CAT AAG ATT CCG AAG CAG A-3′ (reverse); mouse β-actin, 5′-GGC TGT ATT CCC CTC CAT CG-3′ (forward) and 5′-CCA GTT GGT AAC AAT GCC ATGT-3′ (reverse); mouse Gapdh, 5′-AGG TCG GTG TGA ACG GAT TTG-3′ (forward) and 5′-TGT AGA CCA TGT AGT TGA GGT CA-3′ (reverse); mouse Ctgf, 5′-GGG CCT CTT CTG CGA TTT C-3′ (forward) and 5′-ATC CAG GCA AGT GCA TTG GTA-3′ (reverse); mouse Cyr61, 5′-CTG CGC TAA ACA ACT CAA CGA-3′ (forward) and 5′-GCA GAT CCC TTT CAG AGC GG-3′ (reverse); and mouse Ankrd1, 5′-CGA CTC TTG ATG ACC TTC GG-3′ (forward) and 5′-ATT GCT TTG GTT CCA CTC TG-3′ (reverse); mouse Kibra, 5′-TGC TGA GGG AAA CCA AAG CC-3′ (forward) and 5′-CTG GAC CAT AGG TCG GAG TG-3′ (reverse).

Primers used to analyze ChIP results were as follows: intergenic control, 5′-CTG GAA GCA TGG TCT GGT TT-3′ (forward) and 5′-GCA GGG GCA GGT ATA TCA AA-3′ (reverse); *CTGF* promoter region (positive control), 5′-GGA GTG GTG CGA AGA GGA TA-3′ (forward) and 5′-GCC AAT GAG CTG AAT GGA GT-3′ reverse); region YCS2345, 5′- GTA TGC GGC CGA GAG TGA-3′ (forward) and 5′- GCG ACT GCT CCA TCT TCC-3′ (reverse); and region YCS2346, 5′-TCC AAA TAG CCA CTC TGC TTC-3′ (forward) and 5′-TGC AGA ATG TGA TTT GTA TCT TCA-3′ (reverse)

Primers used to genotype mouse lines were as follows: *Sav1*-floxed, 5′-TGG TTT GCT TTT TAG TGG CC-3′ (forward) and 5′-TGC TGG TTT TGT CTC ACT AA-3′ (reverse); *Lats1*-floxed, 5′-TTG TTG CTG GTG TTG TTT CC-3′ (forward) and 5′-ATG AAT GAA CCT GAG GCT GC-3′ (reverse); *Lats2*-floxed, 5′-CCG GAG TCA TTG CTT GTT TT-3′ (forward) and 5′-GGA GAT CCT GGG TAC TGC AC-3′ (reverse); and Albumin-Cre line, 5′-GTG TTG CCG CGC CAT CTG C-3′ (forward) and 5′-CAC CAT TGC CCC TGT TTC ACT ATC-3′ (reverse).

Chromatin immunoprecipitation (ChIP)

Detailed procedure of ChIP after cell harvest is as followed. Harvested cells were lysed in SDS lysis buffer (1% SDS, 10 mM EDTA, 50 mM Tris-Cl pH 8.0) and sonicated using Bioruptor for 15 minutes. Lysate was cleared by centrifugation for 5 minutes at 16,100 × g in 4°C and then 10-fold diluted in Dilution buffer (0.01% SDS, 1.1% Triton X-100, 1.2 mM EDTA, 16 .7 mM Tris-Cl pH 8.0, 167 mM NaCl). Diluted lysate was pre-cleared for immunoprecipitation with 2 µg of IgG and Protein A/G bead(GenDepot) in 4°C for 1 hour with rotation. Pre-cleared lysate was incubated with antibodies for overnight and precipitated using Protein A/G bead for 2 hours in 4°C with rotation. Precipitated beads were washed with Low Salt Wash buffer (0.1% SDS, 1% Triton X-100, 2 mM EDTA, 20 mM Tris-Cl pH 8.0, 150 mM NaCl), High Salt Wash buffer (Low Salt Wash Buffer with 500 mM NaCl), LiCl Wash buffer (0.25 M LiCl, 1% NP40, 1% deoxycholate, 1 mM EDTA, 10 mM Tris-Cl pH 8.0) and two times with TE (10 mM Tris-Cl pH 8.0, 1mM EDTA). DNA/Protein complex was eluted for three times by gentle vortex at RT with Elution buffer (1% SDS, 0.1 M $NaHCO_3$) and then de-crosslinked by incubating at 65 for overnight with 200 mM NaCl. All the buffers described

above were supplemented with protease inhibitors and phosphatase inhibitors as western blot experiments. After RNase and Proteinase K treatment, DNA was purified through Phenol-Chloroform-Isoamyl Alcohol extraction and Ethanol precipitation.

REFERENCES

1. Kim M, Kim M, Lee S, Kuninaka S, Saya H, Lee H, Lee S, Lim DS. cAMP/PKA signalling reinforces the LATS-YAP pathway to fully suppress YAP in response to actin cytoskeletal changes. EMBO J. 2013; 32:1543–1555.
2. Angus L, Moleirinho S, Herron L, Sinha A, Zhang X, Niestrata M, Dholakia K, Prystowsky MB, Harvey KF, Reynolds PA, Gunn-Moore FJ. Willin/FRMD6 expression activates the Hippo signaling pathway kinases in mammals and antagonizes oncogenic YAP. Oncogene. 2012; 31:238–250.
3. Visser-Grieve S, Hao Y, Yang X. Human homolog of Drosophila expanded, hEx, functions as a putative tumor suppressor in human cancer cell lines independently of the Hippo pathway. Oncogene. 2012; 31:1189–1195.
4. Levy D, Adamovich Y, Reuven N, Shaul Y. Yap1 phosphorylation by c-Abl is a critical step in selective activation of proapoptotic genes in response to DNA damage. Mol Cell. 2008; 29:350–361.
